# Successful Treatment of Sjögren's Syndrome Presenting as a Condition Similar to Chronic Capillary Leak Syndrome Using Combination Therapy with High-Dose Intravenous Immunoglobulin and Glucocorticoid

**DOI:** 10.1155/2019/4865024

**Published:** 2019-03-04

**Authors:** Masami Tokura, Tomoko Niwano, Kenji Nagasaka

**Affiliations:** Department of Rheumatology, Ome Municipal General Hospital, Ome, Japan

## Abstract

A 70-year-old woman with Sjögren's syndrome (SS) complained of generalized edema. Computed tomography showed thoracoabdominal fluid, suggesting serositis with SS. 35 mg/day of prednisolone as a monotherapy was ineffective. Moreover, hemoconcentration with hypoalbuminemia without inflammatory signs lead us to consider the systemic capillary leak syndrome (SCLS). Additional treatment with intravenous immunoglobulin (IVIG) and prednisolone dramatically decreased the thoracoabdominal fluid. However, when reducing the prednisolone dose, the thoracoabdominal fluid reincreased. Retreatment with IVIG without increasing the prednisolone dose was ineffective. However, additional prednisolone of 35 mg/day was effective, suggesting SCLS with SS might require combination therapy with IVIG and glucocorticoid.

## 1. Introduction

Systemic capillary leak syndrome (SCLS) is a rare but fatal condition characterized by severe hypotension, hypoalbuminemia, and hemoconcentration [1, 2]. These characteristic symptoms are thought to be caused by excessive vascular permeability due to vascular endothelial dysfunction and leakage of a large amount of plasma component from the blood vessel [2]. Although monoclonal proteinemia is present in about 80% of SCLS [3] and a large number of mediators that promote vascular permeability [4] have been reported, the pathogenesis of SCLS is unknown.

According to these hypotheses, various treatments targeting correction of vascular permeability have been attempted [5–7]. To date, treatment with vascular endothelial cell growth factor (VEGF) inhibitor [5], tumor necrosis factor (TNF)-*α* inhibitor [6], and thalidomide [7] has been attempted, but these have not been widely used. It has been sporadically reported that high-dose intravenous immunoglobulin (IVIG) therapy is effective in some cases of SCLS [8–10]; however, the mechanism of its efficacy is also still unknown.

As for SCLS associated with connective tissue disease (CTD), not only is it rarely encountered in clinical practice, but also there are very few reports [11–15] about it. Because of little information on the treatment strategy for SCLS associated with CTD, it is difficult to draw a conclusion on which therapy should be performed: treatment against SCLS as a vascular event or underlying disease using immunosuppressive agents.

We encountered Sjögren's syndrome (SS) that showed SCLS-like symptoms, from the finding of massive thoracoabdominal fluid and systemic edema with hypoalbuminemia and hematocrit (Ht) level elevation. Repeated episodes wherein combination treatment with glucocorticoid (GC) and IVIG was effective, despite the inefficacy of their monotherapy, may provide a clue on the pathophysiology and treatment strategy of SCLS associated with CTD.

## 2. Case Presentation

A 70-year-old woman complained of systemic edema and excessive weight gain. Since she has hypertension and a history of subarachnoid hemorrhage at the age of 50 years, she had taken antihypertensive agents, including amlodipine besylate and candesartan cilexetil. In year X-25, she was diagnosed with SS because of dry eyes confirmed by the Schirmer and Rose Bengal test, mononuclear cell infiltration around the salivary gland, and the presence of anti-SSA antibodies. In July of year X-1, she visited our hospital due to body weight gain of 3 kg in a month, lower leg edema, and dyspnea. Computed tomography (CT) showed thoracoabdominal fluid. She was admitted in September.

Upon admission, she had normal blood pressure of 119/83 mmHg, and oxygen saturation was 97%. She had no cardiac murmurs. Her respiratory sound attenuates in both lower lungs and marked subcutaneous edema in the abdomen and legs was noted.

Laboratory findings revealed elevated Ht level of 45.6%, with lower total protein (TP) (6.1 g/dL) and albumin (ALB) levels (2.9 g/dL) (Table 1). Thyroid function was normal. Antinuclear antibody showing a centromeric pattern and anti-SSA antibody were positive. Serum M and urinary Bence Jones proteins were not detected. CT showed moderate pleural effusion and ascites. Echocardiography showed small amount of pericardial effusion, no ventricle expansion with normal tricuspid valve systolic pressure gradient, and normal diameter of the inferior vena cava with respiratory fluctuation, indicating that her cardiac function was normal.

**Figure 1:**
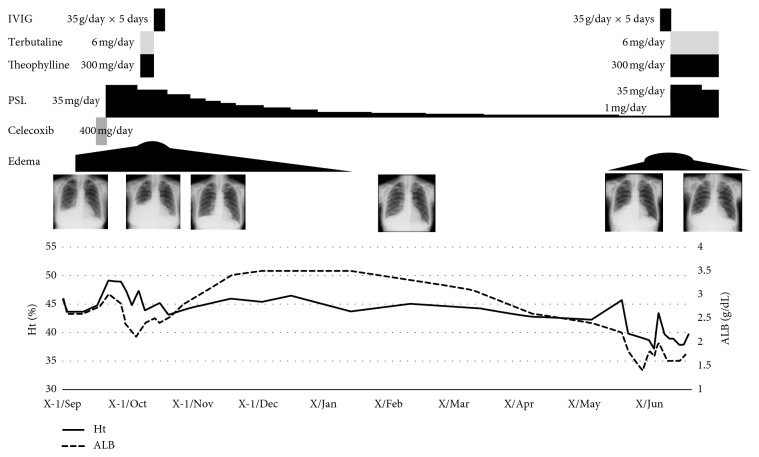
Clinical course of the present case. ALB: albumin; Ht: hematocrit; IVIG: intravenous immunoglobulin; PSL: predonisolone.

At first, we considered the thoracoabdominal fluid as serositis with SS and started celecoxib 400 mg/day (Figure 1). However, pleural effusion did not decrease, and body weight still increased. Prednisolone (PSL) 35 mg/day (0.5 mg/kg/day) was started, and body weight gradually decreased; however, difficulty in breathing, subcutaneous edema, and pleural effusion worsened. In addition, hypoalbuminemia worsened, and the Ht level continued to increase. Other causes resembling SCLS, i.e., angioedema, monoclonal gammopathy of undetermined significance, and CTD other than SS, were distinguished by her clinical history, physical, and laboratory findings. We decided to treat the condition as chronic SCLS, administering terbutaline 6 mg/day and theophylline 300 mg/day starting that day. However, they were ineffective, and hypoalbuminemia progressed. Therefore, 35 g/day (0.4 g/kg/day) of IVIG was administered for 5 days from hospital day 31, in addition to 30 mg/day of PSL. After three days, the Ht level decreased and hypoalbuminemia began to improve. Subcutaneous edema and pleural effusion also improved, and she was discharged.

After discharge, the dose of PSL was gradually decreased. However, after decreasing the PSL dose to 2 mg/day in March of year X, abdominal fullness was noted. Subcutaneous edema and pleural effusion redeveloped and worsened. In May of year X, she had difficulty in breathing and was rehospitalized.

Upon admission, her blood pressure was 102/60 mmHg, and severe abdominal edema was observed. Hemoconcentration, hypoalbuminemia, and thoracoabdominal fluid, as in the previous condition, were also present.

She was diagnosed with relapse of chronic SCLS, and 35 g/day (0.4 g/kg/day) of IVIG was started without increasing the PSL dose because her clinical history suggested that only IVIG might be essential. However, severe abdominal edema did not improve even after 5 days of IVIG therapy. We thought that IVIG alone seemed ineffective. Because the difficulty in breathing worsened, PSL dose was increased to 35 mg/day (0.5 mg/kg/day). Subsequently, shortness of breath, abdominal edema, and thoracoabdominal fluid improved. Hypoalbuminemia also improved. She was then successfully discharged.

## 3. Discussion

This is a rare case of chronic SCLS complicated with SS. Regarding her treatment course, it seems that the combination therapy of moderate dose of PSL and IVIG was effective.

Severe edema and massive thoracoabdominal fluid in this case were considered to be involved in the same mechanism as chronic SCLS. In this case, hypovolemic shock, one of three signs of SCLS, was lacking. However, those symptoms accompanied by hypoproteinemia and hypoalbuminemia indicated excessive vascular permeability with concomitant leakage of plasma components into the extravascular tissue. In addition, repeated similar episodes were also consistent with SCLS [1]. While a typical SCLS [16] rapidly causes systemic edema, hypotension, hemoconcentration, and compartment syndrome, cases of chronic SCLS [17] have been reported recently. These cases were characterized by gradual excessive weight gain and systemic edema. Because this case showed a similar course as that in chronic SCLS, chronic SCLS was mostly considered.

IVIG has been thought to be an effective treatment for SCLS. Various treatments such as VEGF inhibitor [5], TNF-*α* inhibitor [6], and thalidomide [7] have been attempted to correct vascular hyperpermeability, which is considered as a main pathology of SCLS. However, these agents were not widely used. Meanwhile, some reports showed efficacy of IVIG for SCLS [8–10]. Lambert [8] reported that the 5-year survival rate of the IVIG-treated group was 93.8% and that of the non-IVIG group was 67.2%. In a report by Gousseff [9], 4 of 5 patients in the non-IVIG group died, whereas only 1 of 4 patients in the IVIG-treated group died. Therefore, IVIG might be effective for SCLS.

It is sporadically reported that SCLS is also complicated with CTD [11–15]. However, in these cases, GC rather than IVIG was effective, which is different from typical SCLS. As shown in Table 2, SCLS or localized capillary leak syndrome are complicated with SS [11], scleroderma [11–13], polymyositis [11], juvenile dermatomyositis (JDM) [14], and systemic lupus erythematosus (SLE) [15]. In SCLS with CTD, there are 4 treatment patterns: immunosuppressant including GC only (cases 5, 6, 11, and 12), IVIG for GC resistance (case 1), IVIG only (cases 3, 8, 9, and 10), and others (case 2, 7). Although GC seems effective for SCLS with SLE, IVIG is needed for JDM. In contrast, in SCLS with SS, efficacy of GC or IVIG seems different in each case. However, unlike idiopathic SCLS, it could be said that there are SCLS cases with CTD in which GC, not IVIG, showed efficacy.

Anticentromere antibody (ACA) and anti-SSA antibody double-positive SS patients are reported to have higher disease activity [18]. Although the volume of pleural effusion fluctuated, symptoms other than SCLS were not seen during the treatment course, suggesting that SCLS is not related to disease activity of SS in this case. As for anti-SSA antibody, anti-SSA antibody-positive CTD cases with SCLS were reported [11, 15]. Moreover, in the mouse model, anti-Ro 52 antibody has potential to cause salivary gland dysfunction via innate immunity [19]. These facts suggest that anti-SSA antibody might be associated with pathology of SCLS.

In the present case, monotherapy of IVIG or GC was ineffective, but the combined use of both was effective. In the first admission, thoracoabdominal fluid did not decrease with administration of 35 mg/day of PSL, and concomitant use of IVIG was successful. In the second admission, IVIG without increasing the PSL dose did not decrease the thoracoabdominal fluid, but the thoracoabdominal fluid improved after increasing the PSL dose to 35 mg/day when the amount of immunoglobulins seemed to remain in the body. These facts might suggest that not only combination therapy is effective but also the action mechanism of IVIG might be different from that of GC in SCLS with CTD. Otherwise, the pathogenesis of SCLS with CTD may be different from that without CTD.

We reported on SS complicated with SCLS-like symptoms, which was successfully treated with a combination of IVIG and GC. In the case of SCLS with CTD, even if monotherapy of IVIG or GC is ineffective, their combination might be effective. Combination therapy with GC and IVIG should be taken into consideration.

## Figures and Tables

**Table 1:** Laboratory data on first admission.

Peripheral blood		Biochemistry		Immunological test	
WBC	7480 mm^3^	Total protein	6.1 g/dL	IgG	982 mg/dL
Neutrophil	55%	Albumin	2.9 g/dL	IgA	257 mg/dL
Lymphocyte	29.5%	AST	23 U/L	IgM	241 mg/dL
Monocyte	6.6%	ALT	14 U/L	ANA	
Eosinophil	8%	LDH	176 U/L	Centromere pattern	×640
Basophil	0.9%	*γ*-GTP	42 U/L	Anti-SSA antibody	48.2 U/mL
Red blood cell	508 × 10^4^ mm^3^	Blood urea nitrogen	9.2 mg/dL	Anti-SSB antibody	<0.5 U/mL
Hb	14.9 g/dL	Creatinine	0.71 mg/dL	Pleural effusion	
Hematocrit	45.6%	C-reactive protein	0.11 mg/dL	WBC	470 mm^3^
MCV	90 fL	Endocrine		Mononuclear cell	92.2%
Platelet	30.4 × 10^4^ mm^3^	TSH	2.48 *µ*IU/mL	Polynuclear cell	7.8%
		FT3	3 pg/mL	Total protein	3.6 g/dL
		FT4	1.3 ng/dL	LDH	108 U/L

MCV: mean corpuscular volume; AST: aspartate aminotransferase; ALT: alanine aminotransferase; LDH: lactate dehydrogenase; *γ*-GTP: *γ*-glutamyl transpeptidase; TSH: thyroid-stimulating hormone; FT3: free triiodothyronine; FT4: free thyroxine; ANA: antinuclear antibody.

**Table 2:** Characteristics of SCSL with CTD.

Case	Age	Gender	CTD	Treatments before SCLS	Symptoms and/or laboratory findings at the onset of SCLS	Anti-SSA antibody	Treatments for SCLS	Outcome
1 [11]	25	F	SS	GC	Abdominal pain, nausea, vomiting pericardial effusion, ascites	Positive	GC and diuretics-IVIG	Remission
2 [11]	43	M	SS SSc	D-pen	Weight gain, hemoconcentration hypoalbuminemia	Negative	Diuretics and IVIG	Dead
3 [11]	17	M	PM	GC, MTX, AZA	Edema of upper limbs and face, hemoconcentration, hypoalbuminemia	Negative	IVIG	Remission
4 [11]	45	F	SS	None	Oliguria, loss of weight hemoconcentration, hypoalbuminemia	Positive	Transfusion and IVIG	Remission
5 [11]	55	F	SS	None	Hypovolemic shock hemoconcentration, hypoproteinemia	Positive	Transfusion and GC	Remission
6 [12]	61	M	SSc	None	Shortness of breath, profuse sweating, leg edema, severe fatigue, and otalgia	NA	High-dose GC and diuretics	Remission
7 [13]	52	F	SSc	Imatinib mesylate	Facial and lower extremity edema, and elevated alkaline phosphatase	NA	Vasopressor	Dead
8 [14]	7	M	JDM	GC pulse high-dose GC	Edema of the neck and upper limbs, myalgia, oliguria, and hypotension	NA	IVIG	Remission
9 [14]	13	M	JDM	GC pulse	Generalized edema, myalgia, oliguria, hypotension, and tachycardia	NA	IVIG	Remission
10 [14]	3	M	JDM	GC pulse high-dose GC	Edema of the face and neck, oliguria, acute kidney, and respiratory failure	NA	IVIG	Remission
11 [15]	40	F	SLE	GC CPA, AZA	Nausea, vomiting, and intestinal wall edema at CT scan	NA	Transfusion and IS	Remission
12 [15]	41	F	SLE	GC HCQ	Nausea, vomiting, and intestinal wall edema at CT scan	Positive	High-dose GC	Remission

F: female; M: male; NA: not available; SCLS: systemic capillary leak syndrome; CTD: connective tissue disease; SS: Sjögren's syndrome; SSc: systemic sclerosis; PM: polymyositis; JDM: juvenile dermatomyositis; SLE: systemic lupus erythematosus; GC: glucocorticoid; IVIG: intravenous immunoglobulin therapy; D-pen: D-penicillamine; AZA: azathioprine; CPA: cyclophosphamide; HCQ: hydroxychloroquine; MMF: mycophenolate mofetil.
